# Hints for Advanced Genotype–Phenotype Correlation in a Cohort of Suspected Facioscapulohumeral Muscular Dystrophy Patients From Serbia

**DOI:** 10.1111/ene.70764

**Published:** 2026-11-05

**Authors:** Noemi Albano, Branislav Ralic, Andi Nuredini, Riccardo Cuoghi Costantini, Ana Marjanovic, Stojan Peric, Rossella Tupler

**Affiliations:** ^1^ Department of Biomedical, Metabolic and Neural Sciences University of Modena and Reggio Emilia Modena Italy; ^2^ Department of Neurology Clinical Hospital Center Zvezdara Belgrade Serbia; ^3^ Division of Clinical Statistics, Department of Medical and Surgical Sciences of Children and Adults University of Modena and Reggio Emilia Modena Italy; ^4^ Neurology Clinic University Clinical Center of Serbia Belgrade Serbia; ^5^ Faculty of Medicine University of Belgrade Belgrade Serbia

**Keywords:** clinical heterogeneity, facioscapulohumeral muscular dystrophy, FSHD molecular diagnosis, penetrance, standardized phenotyping

## Abstract

**Background:**

Facioscapulohumeral muscular dystrophy (FSHD) is a hereditary myopathy characterized by marked clinical variability and diagnostic complexity. It comprises two forms: FSHD1, associated with contraction of D4Z4 repeats on chromosome 4q35, and FSHD2, linked to variants in chromatin remodeling genes. The presence of FSHD‐associated molecular signatures in healthy individuals further complicates diagnosis.

**Methods:**

We evaluated 39 unrelated Serbian probands with suspected FSHD. Family investigations were conducted in 30 probands (76.9%), involving 54 relatives (total *n* = 93). Clinical assessment was performed using the Comprehensive Clinical Evaluation Form (CCEF), and molecular testing followed the standard workflow for FSHD1.

**Results:**

A D4Z4 reduced allele (DRA, 1–10 repeats) was identified in 31 probands (79.5%). Of these, 24 (77.4%) showed a classical phenotype, while 7 (22.6%) had facial‐sparing presentations. Among 39 relatives from 23 DRA‐positive families, 13 (33.3%) carried a DRA; among them, 5 had classical FSHD and 8 were asymptomatic or mildly affected. Eight probands (20.5%) were DRA‐negative. Among 15 relatives from these families who were all DRA‐negative, one showed a classical phenotype, and six were asymptomatic or mildly affected.

**Conclusion:**

These findings confirm the marked phenotypic heterogeneity of suspected FSHD and highlight the importance of standardized clinical assessment and family investigations to improve diagnosis, genetic counseling, and patient selection for clinical trials.

## Graphical abstract

Analysis of Serbian families affected by FSHD highlights the complexity of genotype–phenotype correlations, characterized by incomplete penetrance and marked intrafamilial heterogeneity. The involvement of relatives in the diagnostic process is essential to ensure accurate counseling and appropriate patient management. A comprehensive baseline clinical characterization associated with genomic studies provides a valuable method for studying genotype–phenotype correlations. Future studies integrating genomic and longitudinal clinical data will support disease stratification and patient selection for testing emerging therapeutic strategies.

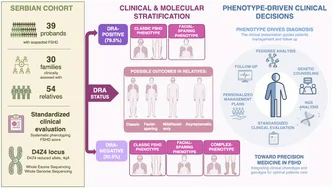

## 1 Introduction

Facioscapulohumeral muscular dystrophy (FSHD) is a hereditary myopathy characterized by progressive, asymmetric atrophy and weakness of the facial, shoulder girdle, upper arms, trunk, and anterior compartment muscles of the lower legs, and high variability in clinical expression [1, 2]. The prevalence of FSHD has been estimated between 1 in 8000 and 1 in 20,000 [3, 4].

The first cases of FSHD were clinically described in the mid‐19th century in France. As early as 1862, Duchenne provided a description of a typical case. He reported that the patient had progressive fatty muscular atrophy beginning between the ages of 5 and 7, initially affecting the face, manifesting as atrophy of muscles around the eyes, lips, and cheekbones. After remaining stationary for a few years, the disease would then progress to the limbs and trunk. The genetic locus responsible for FSHD was mapped in 1990 to chromosome 4q35, and in 1992, contraction in the number of tandemly repeated D4Z4 sequences was identified to segregate with disease in FSHD families [5]. Since then, FSHD has been genetically associated with contractions of the D4Z4 macrosatellite array located at the subtelomeric region of chromosome 4q35 [6]. A pathogenic threshold of 10 or fewer repeat units associated with the 4qA polymorphism is widely applied in molecular diagnostics. Nevertheless, the relationship between genotype and clinical expression is complex [7]. Severe phenotypes are more consistently observed in carriers of very short alleles (1–3 repeats) [8], while individuals with intermediate alleles (6–10 repeats) show wide inter‐ and intrafamilial variability [9, 10], including non‐penetrant DRA carriers and atypical phenotypes [11]. In the FSHD2 disease subtype, patients carry two D4Z4 arrays with more than 10 RUs but bear heterozygous dominant mutations in genes such as *SMCHD1* (Structural Maintenance of Chromosomes flexible Hinge Domain containing‐1), *LRIF1* (Ligand dependent nuclear Receptor Interacting Factor 1), and *DNMT3B* (DNA Methyltransferase 3 beta), encoding epigenetic regulators that might influence the chromatin structure of the D4Z4 locus.

The current pathogenic model for FSHD proposes that D4Z4 contraction or variants in chromatin remodeling genes lead to epigenetic derepression and aberrant expression of the DUX4 retrogene in the context of a permissive 4q haplotype [12]. However, accumulating clinical and epidemiological evidence indicates that this mechanism alone cannot fully explain the variability of disease onset and progression [13]. Large‐scale studies based on standardized clinical classifications have confirmed that FSHD presents with marked phenotypic heterogeneity, even among individuals carrying the same molecular defect in the same family [13, 14]. Moreover, D4Z4 reduced alleles (DRAs) and other molecular signatures of FSHD have also been reported in healthy individuals [14] and in patients with other neuromuscular disorders [7]. Sex has been identified as a potential phenotypic modifier in FSHD. Specifically, female patients tend to have later disease onset, and female relatives often exhibit milder clinical phenotypes compared to their male counterparts [11, 15]. Moreover, disease penetrance and severity are reduced in relatives, and this depends on the degree of kinship with the proband [11]. Overall, FSHD represents an excellent example of a complex disease, in which the interplay between genetic, epigenetic, and environmental factors has a fundamental role in disease expression [16, 17]. Given these complexities, epidemiological investigations in different populations are essential to better define the frequency, clinical spectrum, and penetrance of the molecular haplotype associated with FSHD.

In this study, we analyze a cohort of subjects with suspected FSHD from the Neurology Clinic at the University Clinical Center of Serbia in Belgrade. By applying the Comprehensive Clinical Evaluation Form (CCEF), we provide a systematic stratification of phenotypes, offering insights into disease variability and contributing to a more precise clinical characterization of FSHD in this population. This work represents the first clinical and molecular characterization of FSHD families in Serbia. To date, there is an important gap in the understanding of disease penetrance and variability in the region, as no other large‐scale studies have been reported in this population. Establishing a national reference is essential to improve diagnostic accuracy, guide genetic counseling, and support the integration of Serbian patients into international FSHD research and therapeutic trials. By providing robust baseline data, this study lays the foundation for future longitudinal and translational research efforts in Southeast Europe.

## 2 Materials and Methods

### 2.1 Patient Selection and Sample Collection

The research protocol was approved by the Ethics Committee of the University Clinical Center of Serbia (approval number 1177/10 dated December 28, 2022). Each participant received detailed information regarding the nature of the study and the procedures and signed informed consent. The study included adult individuals presenting with clinical features of FSHD, as well as their family members. Probands were all symptomatic; family members were either symptomatic or completely asymptomatic, unaware of the disease. Participants were initially identified through a search of electronic medical databases Infomedis and Heliant, as well as through the list of outpatient and inpatient records of patients with muscular dystrophy at the Neurology Clinic, University Clinical Center of Serbia, between 2007 and 2024. In both cases, the code for muscular dystrophy per the ICD (International Classification of Diseases) system was used since there is no specific code for FSHD.

Candidates who agreed to participate were reevaluated in an outpatient setting during the period from March 1, 2023, to March 1, 2024. Out of 85 patients with the FSHD phenotype, contact could not be established with 22 individuals due to invalid phone numbers, changes in residence, or nonattendance at follow‐up appointments. Nine patients who initially agreed to participate did not attend their scheduled testing appointments, four declined participation, and six were unable to participate due to advanced age, severe comorbid disabling conditions, or inability to obtain medical referrals from the national health insurance system. In five patients, another neurological diagnosis was established based on results from targeted next‐generation sequencing. At the end, overall 93 individuals (39 index cases, 54 relatives) agreed to participate in the study. There were no significant sociodemographic differences between participants and nonparticipants. In the group of nonparticipants, 73% were males and 27% were females; the mean age was 53.2 (±18.4) years.

### 2.2 Clinical Examination

Each subject recruited during the study was examined by a trained neurologist using the CCEF scale [2]. The CCEF questionnaire is structured into different sections. The Evaluation Form (CCEF Section 1) gathers demographic information, family history, age at symptom onset, motor evaluation, and assessment of muscle strength using the Medical Research Council (MRC) scale. This section is also important for a differential diagnosis with other myopathies, as it emphasizes the presence of both typical and atypical FSHD characteristics. The FSHD score (CCEF Section 2) serves as a numerical tool to quantify muscle weakness and disease severity in FSHD patients. The score is derived by assessing muscle strength and function in six muscle groups typically affected in FSHD: facial muscles (0–2), scapular stabilizers (0–3), upper limbs (0–2), lower limbs (0–2), pelvic girdle (0–5), and abdominal muscles (0–1). The total score ranges from 0 (*asymptomatic individual*) to 15 (*severe muscle weakness and atrophy*). This scoring system was developed by Lamperti et al. [1] as a practical tool to estimate disease severity in this highly variable condition [1]. The CCEF [2] was used to classify all DRA carriers as follows: (1) individuals presenting facial and scapular girdle muscle weakness typical of FSHD (Category A, subcategories A1–A3), (2) individuals with muscle weakness limited to scapular girdle or facial muscles (Category B, subcategories B1 and B2), (3) asymptomatic/healthy individuals (Category C, subcategories C1 and C2), and (4) individuals with myopathic phenotype presenting clinical features not consistent with FSHD canonical phenotype (Category D, subcategories D1 and D2).

Following clinical reassessment, peripheral blood samples were collected in EDTA tubes and subsequently shipped to the Miogen Laboratory in Modena for molecular genetic analysis.

### 2.3 Molecular Characterization

DNA was prepared from peripheral blood mononuclear cells (PBMCs) extracted from fresh‐EDTA blood samples. PBMCs were lysed over the weekend in a solution of 0.5 M pH 8 EDTA, 10% Sarkosyl, and 20 mg/mL of proteinase K enzyme, and subsequently digested with EcoRI, EcoRI/BlnI, and XapI restriction enzymes in order to distinguish 4q from 10q alleles. HindIII digestion was performed for the identification of 4qA/4qB polymorphisms. Digested DNAs were electrophoresed using PULSED‐FIELD GEL ELectrophoresis (PFGE) in 1% agarose gels, as previously described, and by linear 0.4% gel electrophoresis according to established protocols [6, 12]. Allele sizes and the presence or absence of the 4qA allele were estimated by Southern hybridization with the radioactively labeled probes p13E‐11 and qA/qB probes. The results of each hybridization were visualized via autoradiography. In DRA‐negative cases with a persistent clinical suspicion of an inherited myopathy, additional molecular investigations were performed, including whole‐exome sequencing (WES) in five patients, with particular focus on the genes associated with FSHD2, while whole‐genome sequencing (WGS) was additionally performed in one of them to evaluate alternative genetic diagnoses. The extent of WGS was low due to the unavailability of the method in Serbia. Two patients declined further testing.

### 2.4 Statistical Analysis

Descriptive statistics were calculated for demographic and clinical variables. Continuous variables are presented as mean ± standard deviation (SD), and categorical variables as frequencies and percentages. Given the limited sample size, particularly within individual clinical subgroups, regression or multivariate analyses were not considered appropriate, as they would not have provided sufficiently robust estimates. Therefore, the statistical analysis was limited to descriptive statistics appropriate for the available sample size and study design. Analyses were conducted using standard statistical software (e.g., R version 4.3.3).

## 3 Results

We evaluated a cohort of 39 unrelated Serbian individuals referred for a clinical suspicion of FSHD (Figure 1A). A total of 30 probands (76.9%) were clinically assessed together with available family members (Figure 1B). Demographic, clinical, and molecular features of probands and relatives stratified by DRA status are summarized in Table 1. A total of 93 subjects were investigated with combined clinical and molecular evaluation. Molecular analysis identified a D4Z4 reduced allele (DRA; 1–10 repeat units, RUs) in 31 out of 39 probands (79.5%), hereafter referred to as the DRA‐positive group. The remaining eight probands (20.5%) carried D4Z4 alleles within the normal size range and were classified as the DRA‐negative group. Among DRA‐positive probands, 23 out of 31 (74.2%) were evaluated together with 39 relatives; 8 were considered isolated cases (25.8%). In the DRA‐negative group, 7 probands out of 8 (87.5%) were assessed together with 15 relatives.

**FIGURE 1:**
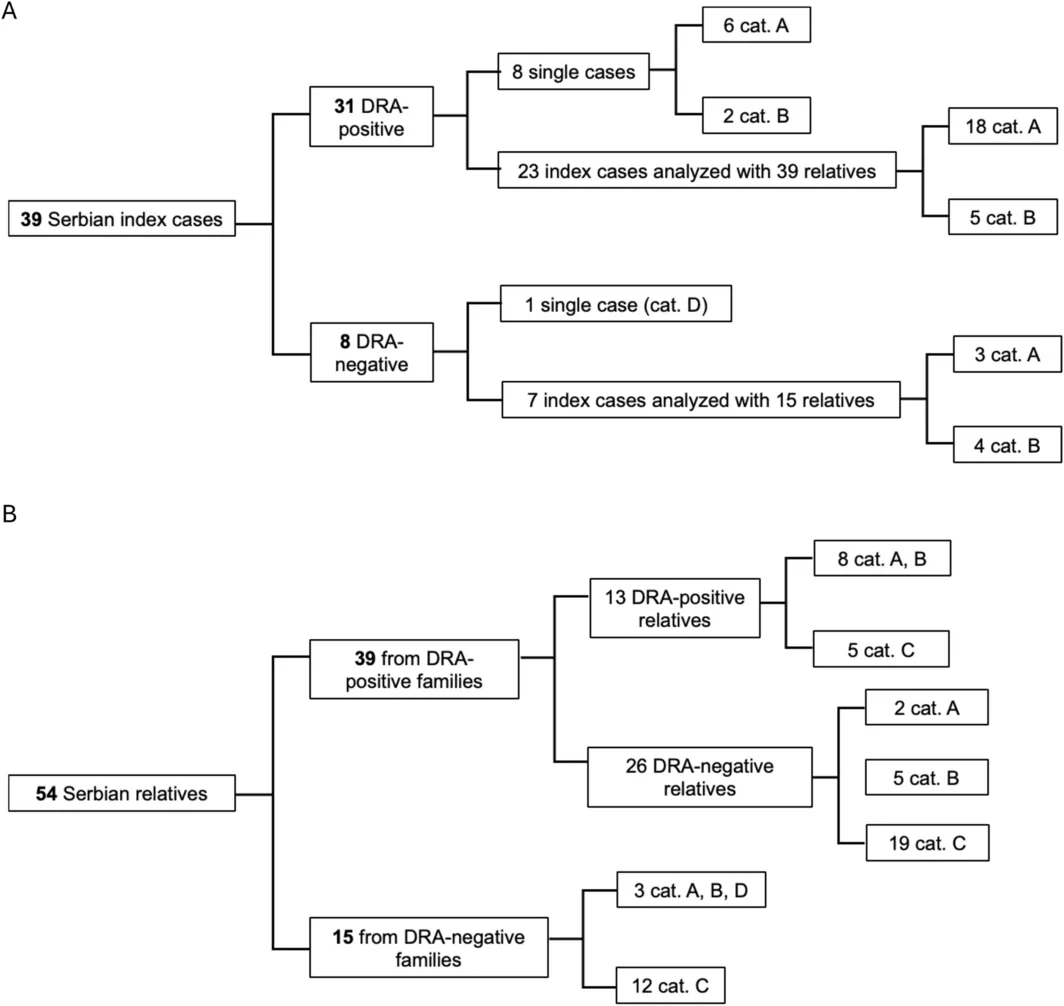
Structure of the cohort in relation to the results of the molecular investigations together with the results of the standardized clinical assessment for index cases (A) and relatives (B). The abbreviation “cat.” stands for CCEF clinical category.

**TABLE 1:** Demographic table of Serbian cohort characteristics.

	Probands (*n* = 39)		Relatives (*n* = 54)	
	DRA‐positive	DRA‐negative	DRA‐positive	DRA‐negative
Sex, *N* (%)				
F	9 (29)	2 (25)	10 (76.9)	21 (51.2)
M	22 (71)	6 (75)	3 (23.1)	20 (48.8)
Age (SD), years	42.2 (±15.6)	38.9 (±15.9)	45.6 (±19.2)	50.5 (±16.4)
Age at onset (SD), years	22 (±11.8)	35 (±16.1)	20.8 (±11.5)	NA
FSHD score (SD)	7.5 (±3.4)	3.5 (±2.6)	2.2 (±1.5)	1.8 (±0.8)
DRA units, *N* (%)				
1–3 U	8 (20.5)		3 (5.6)	
4–6 U	17 (43.6)		8 (14.8)	
7–8 U	3 (7.7)		1 (1.9)	
9–10 U	3 (7.7)		1 (1.9)	
≥ 11 U	8 (20.5)		41 (75.9)	

*Note:* Continuous variables are reported as mean ± standard deviation (SD). Age at onset for DRA‐negative family members was available for only one individual (25 years); mean and SD were not calculated.

### 3.1 Clinical and Molecular Characteristics of DRA‐Positive Probands and Relatives

Figure 1 describes the cohort based on the results of the molecular investigations together with the results of the standardized clinical assessment. The stratification of probands and relatives according to DRA status and clinical phenotype is detailed in Figure 2. In our cohort of 31 DRA‐positive probands, 22 (71%) were males. Mean age at evaluation was 40.7 (±15.2) years for males (95% CI, 34.0–47.4) and 42.7 (±17.6) for females (95% CI, 29.2–56.2). The mean age at onset was 20.5 (±10.9) years for males (95% CI, 15.7–25.4) and 25.6 (±13.8) for females (95% CI, 14.9–36.2). The FSHD score was 6.9/15 (±3.3) for males and 9.1 (±3.4) for females.

**FIGURE 2:**
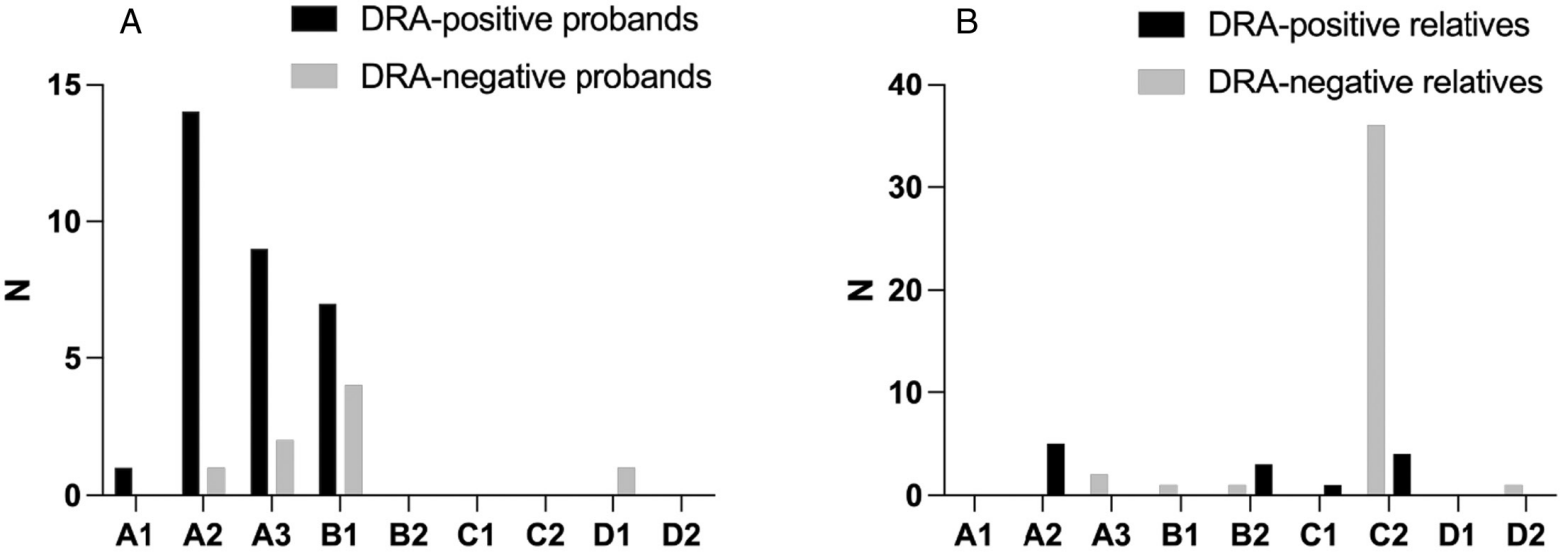
CCEF clinical category distribution for DRA‐positive and DRA‐negative probands (A) and relatives (B).

Stratifying the cohort according to CCEF categories (Figure 2A), 24 out of 31 (77.4%) DRA‐positive probands were classified as Category A, and 7 (22.6%) displayed facial‐sparing phenotypes (Category B1). Of the 39 relatives from 23 unrelated families that were evaluated, 13 (33.3%) were found to be DRA‐positive; 3 were males (23.1%). Five of the DRA‐positive relatives (38.5%) (Figure 2B) were categorized as Category A, three (23%) displayed mild facial weakness (Category B2), and five (38.5%) were asymptomatic. The mean FSHD score for eight symptomatic DRA‐positive relatives was 2.2 (±1.5).

### 3.2 Clinical and Molecular Characteristics of DRA‐Negative Probands and Relatives

Eight probands were found to be DRA‐negative yet displaying muscle weakness suggestive of FSHD. Of these, 7 were evaluated with 15 relatives. The mean age at evaluation for probands was 38 years (±15.8) (95% CI, 24.7–51.2), and the mean age at onset was 35.0 (±16.1) (95% CI, 24.8–51.2). The mean FSHD score was assessed as 3.5 (±2.5). The mean FSHD score for five symptomatic DRA‐negative relatives was 1.8 (±0.8). Four out of eight DRA‐negative probands were classified as Category B1 (44.4%), showing facial‐sparing presentations with relatively late onset (e.g., SF08/01 at age 61; SF36/01 at age 45). One female proband (SF40/01) exhibited an early onset at age 13 despite an incomplete B1 phenotype. Three out of eight probands (SF18/01, SF33/01, and SF42/01) showed classical phenotypes (A2/A3) with onset in adolescence or early adulthood, and one male (SF09/01) affected by myotonic dystrophy Type 1 presented a complex phenotype (D1) with shoulder girdle involvement and prominent asymmetric scapular winging.

### 3.3 Intra‐Familial Phenotypic Heterogeneity of Serbian Families

Families were stratified according to DRA status and the presence of relatives for clinical evaluation (Figure 3). In DRA‐negative families (Figure 3B) with category A index cases, five relatives from three families were asymptomatic and showed no signs of muscle weakness at clinical evaluation. In the case of B1 probands, one family is composed of three relatives classified as Categories A, C, and D, one family with Category B and C relatives, and two families with Category C2 relatives. Among families where the DRA segregates (Figure 3A), 18 out of 23 probands (78.3%) displayed a classical phenotype, and relatives were listed as Category A only in three families (16.7%). The remaining relatives exhibited milder or discordant phenotypes, including isolated facial weakness (B2) or complete absence of clinical signs despite carrying the same DRA. Indeed, in two families, DRA‐positive relatives remained asymptomatic at the time of evaluation (Table 2), at 52 and 61 years of age at testing, respectively. One family, a first‐degree carrier relative (daughter), displayed a classical but milder phenotype (FSHD score 2, cat. A2) if compared to the proband, whereas her older brother exhibited mild facial involvement (FSHD score 1, cat. B2) despite lacking the familial DRA.

**FIGURE 3:**
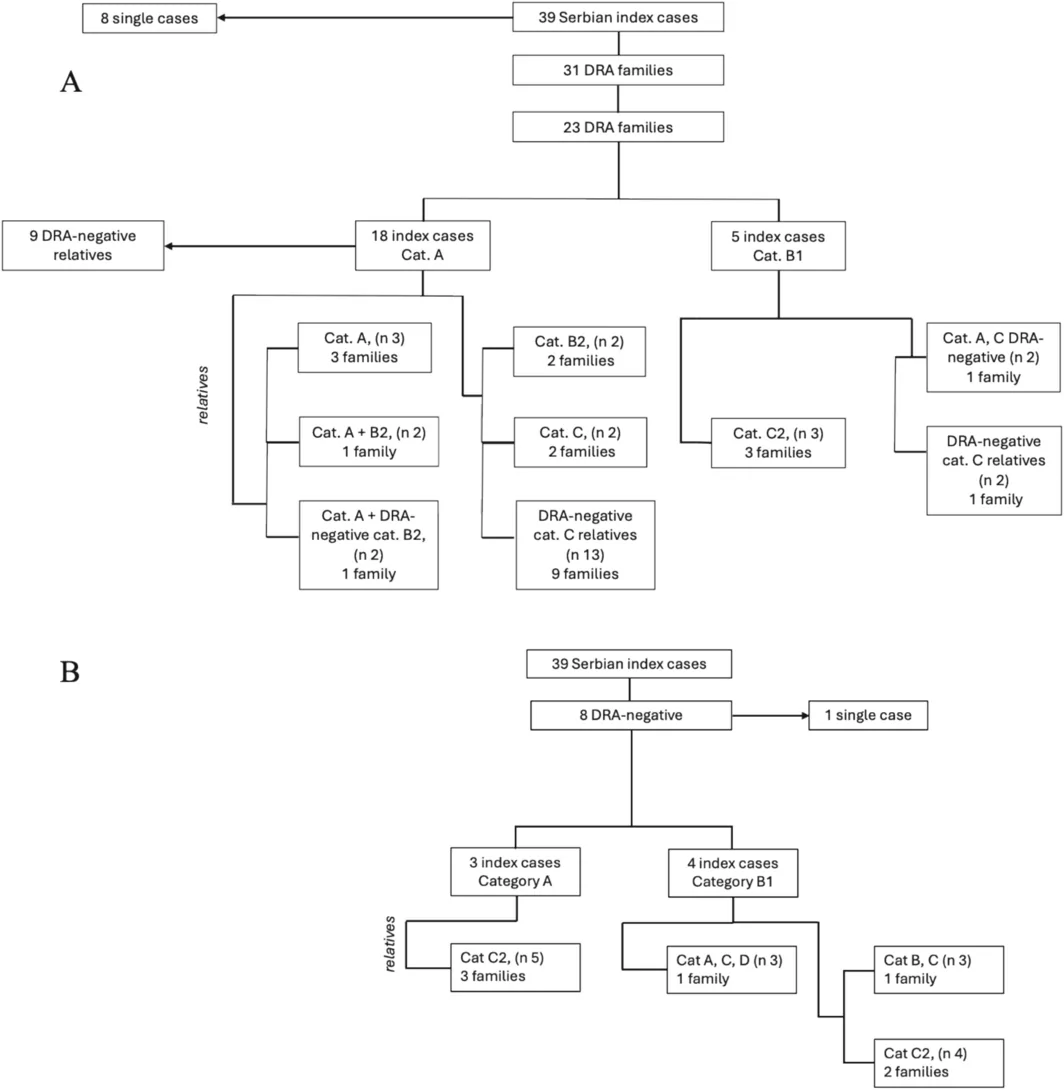
(A) Distribution of the clinical categories observed among 15 relatives of 7 families clinically confirmed as FSHD with no detectable molecular signature, according to the CCEF clinical category (Cat.) of the index cases. The total number of families in each group is indicated, as well as the total number of individuals examined (*n*). (B) Distribution of the clinical categories observed among relatives of 23 families in which a DRA segregates, according to the clinical category of the index cases. The total number of families in each group is indicated, as well as the total number of individuals examined (*n*).

**TABLE 2:** Description of Serbian families with incomplete penetrance and variable clinical expression.

Family	Sex	DRA RUs (kb)	CCEF score category	Proband/relative	Age at testing (years)	Age at onset (years)	Onset site
SF17/01	M	2 (14)	Score 12, A2	Proband	22	17	Distal upper limb, distal lower limb
SF17/02	M	2 (14)	Score 1, B2	Father	61	Asymptomatic	NA
SF02/01	M	3 (17)	Score 2, A3	Proband	21	17	Shoulder girdle
SF02/02	M	3 (17)	Score 4, A2	Brother	21	14	Shoulder girdle
SF26/01	M	3 (19)	Score 6, A2	Proband	29	21	Shoulder girdle
SF26/03	F	3 (19)	Score 5, A2	Mother	58	16	Face
SF22/01	F	4 (21)	Score 9, A2	Proband	56	19	Shoulder girdle
SF22/02	F	4 (21)	Score 2, A2	Daughter	35	Asymptomatic	NA
SF44/01	F	4 (21)	Score 10, A2	Proband	79	50	Pelvic girdle
SF44/02	F	4 (21)	Score 2, A2	Son	38	Asymptomatic	NA
SF07/01	M	4 (22)	Score 5, B1	Proband	29	18	Shoulder girdle, distal upper limb
SF07/02	F	4 (22)	Score 0, C2	Mother	61	Asymptomatic	NA
SF20/01	F	5 (25)	Score 12, A2	Proband	38	15	Shoulder girdle
SF20/02	F	5 (25)	Score 0, C1	Mother	52	Asymptomatic	NA
SF14/01	M	6 (27)	Score 8, A2	Proband	58	18	Shoulder girdle
SF14/03	F	6 (27)	Score 1, B2	Daughter	18	Asymptomatic	NA
SF14/04	F	6 (27)	Score 2, A2	Daughter	21	15	Shoulder girdle
SF05/01	M	7 (30)	Score 2, A3	Proband	43	15	Pectoralis weakness
SF05/03	F	7 (30)	Score 1, B2	Mother	69	Asymptomatic	NA
SF27/01	M	7 (30)	Score 8, B1	Proband	65	26	Shoulder girdle, Distal lower limb, Distal upper limb
SF27/02	M	7 (30)	Score 0, C2	Son	31	Asymptomatic	NA
SF21/01	M	8 (34)	Score 3, B1	Proband	40	17	Shoulder girdle
SF21/03	F	8 (34)	Score 0, C2	Sister	41	Asymptomatic	NA
SF37/01	M	9 (38)	Score 8, A2	Proband	38	8	Distal upper limb
SF37/04	F	9 (38)	Score 0, C2	Mother	75	Asymptomatic	NA

*Note:* Families are identified as Serbian family (SF) and a progressive number. “/01” refers to the proband, and “/other number” refers to the relatives in the same family. Relatives are enumerated sequentially. Onset site was not reported for those subjects.

Abbreviation: NA, not available.

## 4 Discussion

### 4.1 Clinical Heterogeneity and Incomplete Penetrance in the Serbian FSHD Cohort

In our cohort, 31 out of 39 probands (79.5%) with a phenotype suggestive of FSHD carried a DRA. Although the majority of DRA‐positive probands exhibited a classical FSHD phenotype, CCEF Category A (24/31, 77.4%), a substantial proportion showed facial‐sparing presentations, CCEF Category B (7/31, 22.6%), confirming that phenotypic variability is also present among index cases.

Phenotypic variability was even more pronounced among relatives. A total of 13 relatives from DRA‐positive families out of 39 (33.3%) carried the familial DRA. Of these, five (38.5%) exhibited a classical FSHD phenotype; the remaining DRA‐positive relatives either showed isolated signs of subtle facial weakness or were completely asymptomatic at the time of evaluation. These findings provide evidence of incomplete penetrance and variable expressivity, even among adult carriers ranging from 31 to 75 years of age, and point out the limited predictive value of the DRA alone in determining disease manifestation in relatives and offspring. From a clinical standpoint, this represents one of the major challenges in FSHD diagnosis and genetic counseling.

### 4.2 Intra‐Familial Variability Beyond DRA Size

This Serbian cohort displays marked intrafamilial discordance between genotype and phenotype. Probands were predominantly classified as CCEF category A with FSHD scores ranging between 2 and 15, whereas first‐degree relatives frequently displayed milder phenotypes or remained asymptomatic even in mid‐to‐late adulthood (FSHD scores between 0 and 5). Families such as SF5, SF7, SF20, and SF37 exemplify this reduced penetrance, with older relatives remaining clinically unaffected despite carrying the same DRA as the proband. Since first‐degree relatives in these families are older than the probands and remain asymptomatic, it is plausible that they will never develop an overt muscular disease [18]. These observations are consistent with data from larger cohorts from the Italian National Registry [9, 10, 11, 19], supporting the concept that factors beyond D4Z4 may substantially contribute to disease penetrance and expressivity [14, 17]. Indeed, previous studies have suggested that D4Z4 contraction may generate a subclinical sensitized state, in which additional genetic, epigenetic, or environmental modifiers could contribute to the development of an overt myopathy [17, 20]. Although our observations are consistent with this hypothesis, the present study was not designed to investigate these mechanisms. Our findings, while they need to be confirmed in larger cohorts, further support that DRA size alone is not sufficient to predict clinical severity.

A diagnosis of FSHD based solely on molecular findings risks oversimplifying a biologically complex condition. Variants in genes such as *SMCHD1*, a key chromatin repressor of D4Z4, as well as in other epigenetic regulators including *DNMT3B* and *LRIF1*, have been shown to influence the expression of D4Z4 nearby genes in specific genetic contexts [17, 20, 21]. Systematic family‐based studies, including the clinical evaluation of both affected and unaffected carriers of the same allele, may represent a valuable strategy for identifying genetic and epigenetic modifiers that modulate penetrance and expressivity in FSHD. Literature reports multiple examples of incomplete penetrance, variable expressivity, and co‐occurrence of additional genetic conditions (“double” or “triple trouble”) in FSHD [7, 22, 23], emphasizing a complexity that requires particular caution in clinical diagnosis and prognostic assessment.

Figure 3 illustrates the architecture of both DRA‐positive and DRA‐negative FSHD families in terms of phenotypic categories, offering a comprehensive overview of the remarkable clinical diversity observed within and across families. An additional clinically relevant aspect of this cohort is represented by the eight probands (20.5%) lacking a DRA and who nevertheless displayed FSHD‐like clinical presentations (Table 3). In this group, besides the classical presentations, facial‐sparing (Category B1) and complex phenotype (Category D1) predominated, further highlighting the diagnostic challenges posed by atypical and incomplete clinical forms. Extensive genetic investigations, including WES and WGS when available, failed to identify alternative molecular diagnoses in most cases, excluding the presence of pathogenic variants in FSHD2 genes. Possible explanations proposed in the literature include epigenetic dysregulation of the D4Z4 locus, or yet‐unknown genetic mechanisms affecting chromatin structure and gene expression. These hypotheses could not be evaluated in the present study. From a clinical perspective, they emphasize the risk of misdiagnosis when molecular data are interpreted in isolation. Considering all this, it must be acknowledged that the clinical expression of hereditary myopathies is much more heterogeneous than would be expected based on molecular findings. A possible example can be deduced from the recruitment process illustrated in Figure 4. In the case of five subjects initially considered eligible for the FSHD study and subsequently excluded because alternative diagnoses were made based on NGS results. In such scenarios, deep and standardized phenotyping proves more informative than molecular testing alone for achieving a correct diagnosis.

**TABLE 3:** Phenotypes of probands displaying an FSHD‐like phenotype and a normal range of D4Z4 alleles.

Family	Sex	CCEF score category	Age (years)	Age at onset (years)	Onset site	Further diagnostic procedures
SF08/01	F	3, B1	64	61	NA	WES negative
SF09/01	M	8, D1	55	43	Shoulder girdle	WES negative. Positive for myotonic dystrophy Type 1 with severe asymmetric scapular winging.
SF010/01	F	1, B1	27	14	NA	WES negative
SF18/01	M	6, A2	34	26	Shoulder girdle	WES negative, WGS negative
SF33/01	M	2, A3	39	31	Shoulder girdle	WES showed one likely pathogenic and one VUS in the *TTN* gene.
SF36/01	F	2, B1	46	45	Shoulder girdle	WES negative
SF40/01	F	1, B1	14	13	NA	Patient was not interested in further testing.
SF42/01	F	5, A3	31	23	Shoulder girdle	Patient was not interested in further testing.

*Note:* Onset site was not reported for those subjects.

Abbreviation: NA, not available.

**FIGURE 4:**
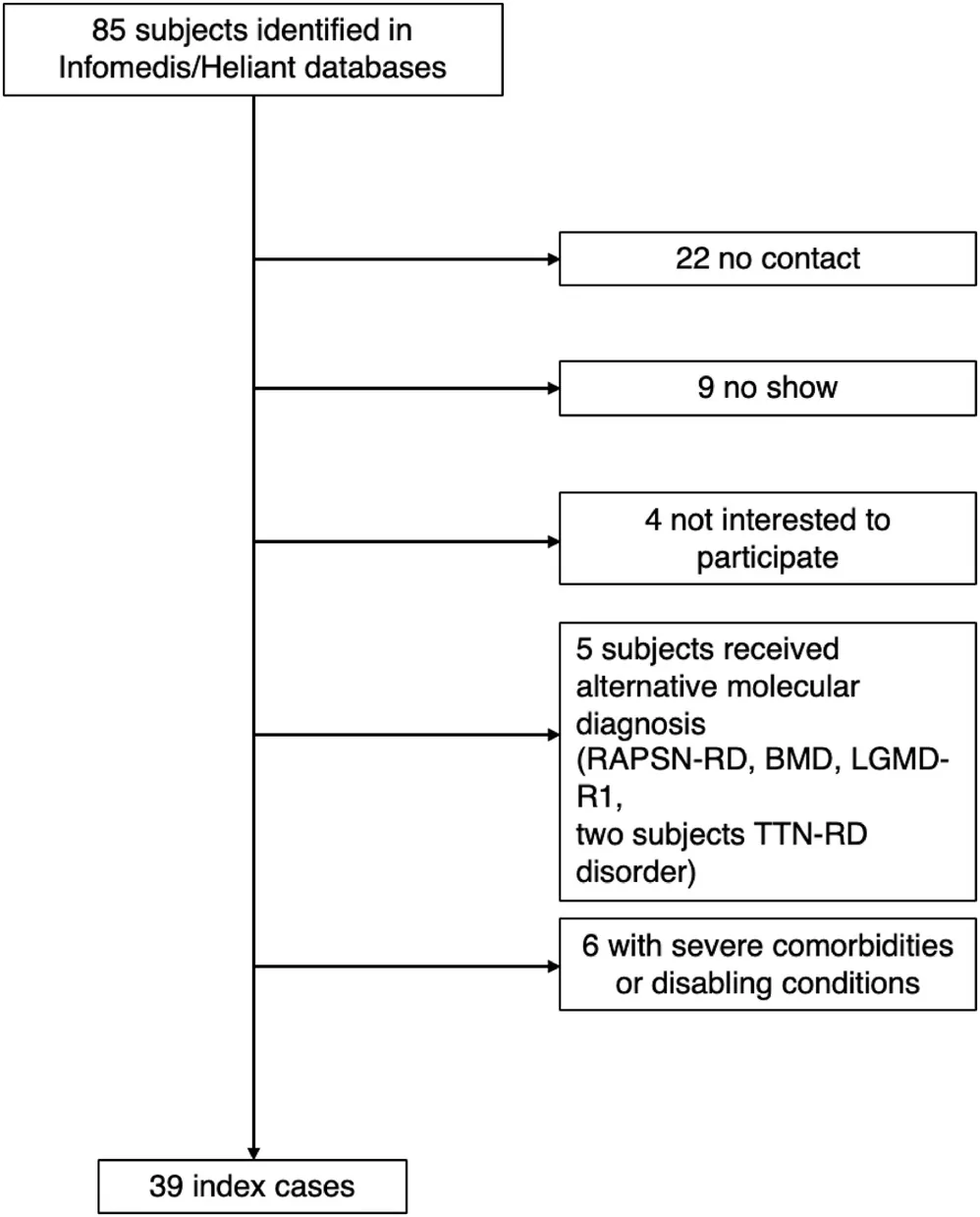
Flowchart of Serbian patients' selection from databases.

### 4.3 Clinical Implications: Beyond a Genotype‐First Approach in FSHD and Neuromuscular Disorders

The widespread availability of molecular testing has progressively shifted neuromuscular diagnostics toward a genotype‐first approach. In FSHD, this paradigm has proved particularly problematic. The molecular signature of FSHD, namely the presence of a DRA on a permissive haplotype, is present in up to 3% of the general population [14], substantially limiting its role as a diagnostic marker. This issue becomes especially critical in carriers of borderline alleles, where the risk of coincidental association between genotype and clinical presentation is high. Large cohort studies have clearly shown that the presence of a D4Z4 contraction does not directly translate into a classical FSHD phenotype [23, 24]. In the study by Ricci et al. [10], only 54.5% of index cases displayed a typical FSHD presentation with combined facial and scapular weakness, while 20.1% showed incomplete phenotypes characterized by isolated facial or scapular girdle involvement; 6.7% displayed minor signs without functional motor impairment; and 18.7% of index cases showed more complex phenotypes with atypical clinical features. These data highlight the limitation of genotype‐first interpretation in clinical practice. To deal with this challenge and to assess the meaning of the presence of a DRA, accurate and standardized phenotyping of the proband and his relatives is essential to reduce the risk that the identification of a DRA represents a fortuitous finding rather than a factor contributing to disease manifestation.

Beyond diagnosis, phenotype carries a stronger prognostic value than genotype in FSHD. Different clinical phenotypes are associated with distinct trajectories of disease progression, functional impairment, and respiratory or extra‐muscular involvement, as shown by Vercelli et al. [19]. The growing number of promising disease‐modifying clinical trials in FSHD highlights the importance of appropriate patient stratification. It must be considered that different phenotypes might be associated with distinct rates of disease progression and functional decline. In this case, robust phenotypic classification may improve patient selection and facilitate the interpretation of the outcomes of clinical trials. Indeed, eligibility criteria based on molecular testing alone risk enrolling a clinically heterogeneous population, potentially reducing the sensitivity to detect. A shift of paradigm toward phenotype‐driven stratification, integrated with molecular data and family‐based interpretation, has become crucial for improving diagnostic accuracy, prognostic assessment, and the design of future successful clinical trials in FSHD. Figure 5 illustrates a structured diagnostic scheme integrating clinical phenotype, DRA status, and family‐based evaluation. The workflow emphasizes the pivotal role of familial studies in interpreting DRA penetrance, distinguishing between familial and sporadic cases, and guiding further genetic investigations, particularly in atypical or DRA‐negative patients.

**FIGURE 5:**
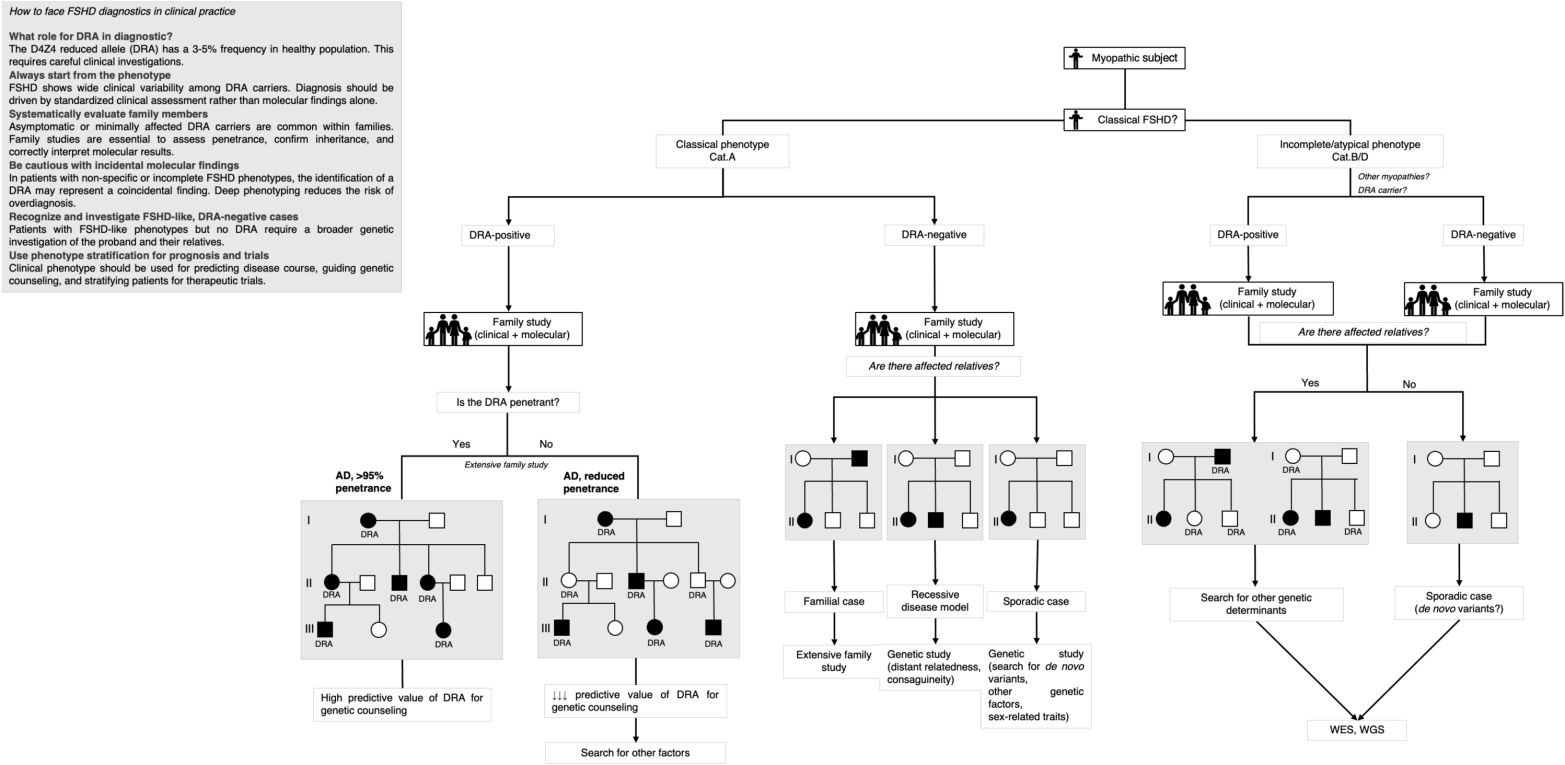
Diagnostic workflow and decision‐making pipeline for facioscapulohumeral muscular dystrophy (FSHD). The investigation starts with a patient displaying a muscle disease. The neurologist evaluates whether the patient exhibits a classical FSHD phenotype (CCEF‐Cat. A) or an incomplete/atypical phenotype (CCEF‐Cat. B/D). Path 1: Classical phenotype, Cat. A, DRA‐positive. Family study. Evaluation of DRA penetrance: All affected relatives are DRA‐positive; the DRA is considered to have high predictive value in the field of genetic counseling. If there are relatives who are unaffected or only minimally affected, the DRA has a lower predictive value. *DRA‐negative*: Check to see if other family members have a similar phenotype. If two affected siblings exhibit similar phenotypes and parents are unaffected, a recessive disease model can be suspected; investigate distant relatedness or consanguinity. In sporadic cases with no family history for neuromuscular diseases, it is essential to search for de novo gene variants, sex‐related traits, or other genetic factors to explain the phenotype. Path 2: Incomplete/Atypical phenotype, Cat. B/D. DRA‐positive: When the DRA segregates in the family, but phenotypes do not agree with the molecular results, it is necessary to investigate other genetic factors. DRA‐negative: When the proband is DRA‐negative with or without myopathic relatives, proceed to WES or WGS to search for other genetic determinants. AD, autosomal dominant; Cat. A, Category A classical FSHD phenotype involving facial and scapular girdle weakness; Cat. B/D, Categories B and D, defining subjects with incomplete phenotypes (facial sparing or exclusive facial muscle weakness) and complex phenotypes, presenting atypical symptoms requiring differential diagnosis with other myopathies; CCEF, Comprehensive Clinical Evaluation Form; Complete penetrance: all DRA carriers present clinical symptoms of FSHD; DRA, D4Z4 Reduced Allele; DRA‐positive/DRA‐negative, indicate the presence/absence of one D4Z4 array of 10 or fewer repeats; Family study, comprehensive clinical assessment and the molecular screening of probands and relatives to assess penetrance and inheritance pattern of the myopathic phenotype; Reduced penetrance: only some DRA carriers exhibit symptoms of the disease; WES, whole‐exome sequencing; WGS, whole‐genome sequencing.

In this regard, the present study provides the first systematic clinical and molecular characterization of FSHD families in Serbia and contributes data from a previously underrepresented population. Although the relatively limited cohort size, particularly after subgroup stratification, restricts the statistical power for formal genotype–phenotype analyses, the use of standardized clinical assessment alongside comprehensive family‐based genetic evaluation provides a detailed descriptive characterization of FSHD variability in this population. Statistical underpower due to the limited number of cases was the reason why we did not apply classical statistical tests; thus, our results and discussion are mostly descriptive but still useful for understanding genotype–phenotype correlations in FSHD. These findings should be interpreted cautiously, serving as observations that may help generate hypotheses for future studies in larger cohorts. By applying standardized clinical tools alongside genetic testing, we identified patterns of incomplete penetrance and phenotypic variability that may otherwise remain unrecognized. These findings support the importance of integrated diagnostic approaches and may facilitate future collaborative studies aimed at improving access to diagnosis, counseling, and emerging therapies in the region.

## 5 Limitations

Several limitations of this study should be acknowledged. First, although this is the largest Serbian FSHD cohort systematically characterized to date, the overall sample size remains limited, particularly for specific clinical subgroups, which may have reduced the statistical power to fully explore genotype–phenotype associations within individual categories. Nevertheless, considering that the University Clinical Center of Serbia (UCCS) is a tertiary referral center covering a population of approximately 2.5 million people, and based on the estimated prevalence of FSHD, approximately 125–312 affected individuals would be expected within this population. Thus, the 52 FSHD cases (comprising both DRA‐positive and DRA‐negative individuals with a phenotype different than C1 and C2) included in our study account for more than 50% of the expected cases, supporting the representativeness of our cohort despite its relatively small overall size. Second, due to the retrospective nature of the study and the inclusion of patients identified through a diagnostic database collected between 2007 and 2024, a potential selection bias cannot be excluded. Although the patients included in the cohort were identified through our diagnostic pathway, their participation in clinical and molecular evaluations required the ability and willingness to attend visits at our center. Therefore, patients who were able or motivated to undergo regular evaluation may be overrepresented, while those who did not attend follow‐up visits, for example because of geographical, socioeconomic, or other barriers, may be underrepresented. As a result, the patients included in our cohort may not fully represent the entire spectrum of FSHD phenotypes in the general population, and referral patterns, availability of molecular testing, and access to regular follow‐up may have influenced cohort composition. Third, clinical phenotyping was performed according to the 2016 CCEF classification [2], which was the reference framework at the time of study design and data collection. The recently introduced CCEF‐R [25] represents a refinement of this classification and may further facilitate the harmonization of clinical assessments across future FSHD cohorts. In this regard, the original CCEF part A considers information regarding family geographical origins, education, profession, clinical history, physical activities, and reproduction that can be used for epidemiological studies.

Finally, molecular characterization of DRA‐negative individuals was not complete in all cases. D4Z4 methylation analysis was not performed, and additional genomic investigations, including whole‐exome or WGS, were available only for a subset of patients. Therefore, the possibility of alternative molecular diagnoses or FSHD2‐related mechanisms cannot be completely excluded.

Although we acknowledge that the characterization of epigenetic regulation may represent an additional layer for interpretation of genotype–phenotype correlation, we do not consider currently available protocols for methylation analysis sufficiently reliable for proper assessment and interpretation [26]. Despite these limitations, this study provides a systematic application of clinical phenotypic stratification in a genetically and clinically underrepresented population and supports its importance.

## 6 Conclusion

Our analysis of Serbian FSHD families highlights the complexity of genotype–phenotype correlations, characterized by incomplete penetrance and marked intrafamilial heterogeneity. These findings confirm previous observations while expanding the phenotypic spectrum of FSHD in a new population. From a clinical perspective, inclusion of relatives in the diagnostic work‐up is essential for accurate counseling and patient management. Given the marked clinical heterogeneity and variable disease trajectories, comprehensive baseline clinical characterization remains highly informative and represents a valid approach for investigating genotype–phenotype correlations.

Future studies integrating genomic, epigenetic, and longitudinal clinical data will be critical to improve disease stratification and optimize selection of patients for emerging therapeutic strategies.

## Author Contributions

**Noemi Albano:** conceptualization, investigation, writing – original draft, data curation, formal analysis. **Branislav Ralic:** investigation, conceptualization, writing – original draft, data curation, formal analysis. **Andi Nuredini:** writing – review and editing. **Riccardo Cuoghi Costantini:** formal analysis, writing – review and editing, methodology. **Ana Marjanovic:** resources, data curation, visualization. **Stojan Peric:** investigation, supervision, writing – review and editing. **Rossella Tupler:** conceptualization, writing – review and editing, methodology, supervision, formal analysis.

## Funding

The authors have nothing to report.

## Ethics Statement

The research protocol was approved by the Ethics Committee of the University Clinical Center of Serbia (approval number 1177/10, dated December 28, 2022).

## Consent

The participants provided their written informed consent to participate in this study.

## Conflicts of Interest

The authors declare no conflicts of interest.

## Data Availability

The data that support the findings of this study are available on request from the corresponding author. The data are not publicly available due to privacy or ethical restrictions.

## References

[1] C. Lamperti , G. Fabbri , L. Vercelli , et al., “A Standardized Clinical Evaluation of Patients Affected by Facioscapulohumeral Muscular Dystrophy: The FSHD Clinical Score,” Muscle & Nerve 42 (2010): 213–217. 10.1002/mus.21671 20544930

[2] G. Ricci , L. Ruggiero , L. Vercelli , et al., “A Novel Clinical Tool to Classify Facioscapulohumeral Muscular Dystrophy Phenotypes,” Journal of Neurology 263 (2016): 1204–1214. 10.1007/s00415-016-8123-2 PMC4893383 27126453

[3] M. Mostacciuolo , E. Pastorello , G. Vazza , et al., “Facioscapulohumeral Muscular Dystrophy: Epidemiological and Molecular Study in a North‐East Italian Population Sample,” Clinical Genetics 75 (2009): 550–555. 10.1111/j.1399-0004.2009.01158.x 19320656

[4] J. C. W. Deenen , H. Arnts , S. M. van der Maarel , et al., “Population‐Based Incidence and Prevalence of Facioscapulohumeral Dystrophy,” Neurology 83 (2014): 1056–1059. 10.1212/WNL.0000000000000797 PMC4166358 25122204

[5] C. Wijmenga , J. E. Hewitt , L. A. Sandkuijl , et al., “Chromosome 4q DNA Rearrangements Associated With Facioscapulohumeral Muscular Dystrophy,” Nature Genetics 2 (1992): 26–30. 10.1038/ng0992-26 1363881

[6] R. J. L. F. Lemmers , P. de Kievit , L. Sandkuijl , et al., “Facioscapulohumeral Muscular Dystrophy Is Uniquely Associated With One of the Two Variants of the 4q Subtelomere,” Nature Genetics 32 (2002): 235–236. 10.1038/ng999 12355084

[7] G. Ricci , M. Zatz , and R. Tupler , “Facioscapulohumeral Muscular Dystrophy: More Complex Than It Appears,” Current Molecular Medicine 14 (2014): 1052–1068. 10.2174/1566524014666141010155054 PMC4264243 25323867

[8] A. Nikolic , G. Ricci , F. Sera , et al., “Clinical Expression of Facioscapulohumeral Muscular Dystrophy in Carriers of 1–3 D4Z4 Reduced Alleles: Experience of the FSHD Italian National Registry,” BMJ Open 6 (2016): e007798. 10.1136/bmjopen-2015-007798 PMC4716236 26733561

[9] L. Ruggiero , F. Mele , F. Manganelli , et al., “Phenotypic Variability Among Patients With D4Z4 Reduced Allele Facioscapulohumeral Muscular Dystrophy,” JAMA Network Open 3 (2020): e204040. 10.1001/jamanetworkopen.2020.4040 PMC7195625 32356886

[10] G. Ricci , F. Mele , M. Govi , et al., “Large Genotype–Phenotype Study in Carriers of D4Z4 Borderline Alleles Provides Guidance for Facioscapulohumeral Muscular Dystrophy Diagnosis,” Scientific Reports 10 (2020): 21648. 10.1038/s41598-020-78578-7 PMC7730397 33303865

[11] G. Ricci , I. Scionti , F. Sera , et al., “Large Scale Genotype–Phenotype Analyses Indicate That Novel Prognostic Tools Are Required for Families With Facioscapulohumeral Muscular Dystrophy,” Brain 136 (2013): 3408–3417. 10.1093/brain/awt226 PMC3808686 24030947

[12] R. J. L. F. Lemmers , P. J. van der Vliet , R. Klooster , et al., “A Unifying Genetic Model for Facioscapulohumeral Muscular Dystrophy,” Science 329 (2010): 1650–1653. 10.1126/science.1189044 PMC4677822 20724583

[13] V. Salsi , G. N. A. Vattemi , and R. G. Tupler , “The FSHD Jigsaw: Are We Placing the Tiles in the Right Position?,” Current Opinion in Neurology 36 (2023): 455–463. 10.1097/WCO.0000000000001176 PMC10487374 37338810

[14] I. Scionti , F. Greco , G. Ricci , et al., “Large‐Scale Population Analysis Challenges the Current Criteria for the Molecular Diagnosis of Fascioscapulohumeral Muscular Dystrophy,” American Journal of Human Genetics 90 (2012): 628–635. 10.1016/j.ajhg.2012.02.019 PMC3322229 22482803

[15] P. Sakellariou , K. Kekou , H. Fryssira , et al., “Mutation Spectrum and Phenotypic Manifestation in FSHD Greek Patients,” Neuromuscular Disorders 22 (2012): 339–349. 10.1016/j.nmd.2011.11.001 22357364

[16] V. Salsi , F. Losi , B. Fosso , et al., “Nucleolar FRG2 lncRNAs Inhibit rRNA Transcription and Cytoplasmic Translation, Linking FSHD to Dysregulation of Muscle‐Specific Protein Synthesis,” Nucleic Acids Research 53 (2025): gkaf643. 10.1093/nar/gkaf643 PMC12242771 40637237

[17] V. Salsi , F. Losi , M. Salani , P. D. Kaufman , and R. Tupler , “Posttranscriptional RNA Stabilization of Telomeric RNAs FRG2, DBE‐T, D4Z4 at Human 4q35 in Response to Genotoxic Stress and D4Z4 Macrosatellite Repeat Length,” Clinical Epigenetics 17 (2025): 73. 10.1186/s13148-025-01881-5 PMC12049803 40320530

[18] S. Sacconi , P. Camaño , J. C. de Greef , et al., “Patients With a Phenotype Consistent With Facioscapulohumeral Muscular Dystrophy Display Genetic and Epigenetic Heterogeneity,” Journal of Medical Genetics 49 (2012): 41–46. 10.1136/jmedgenet-2011-100101 PMC3560331 21984748

[19] L. Vercelli , F. Mele , L. Ruggiero , et al., “A 5‐Year Clinical Follow‐Up Study From the Italian National Registry for FSHD,” Journal of Neurology 268 (2021): 356–366. 10.1007/s00415-020-10144-7 PMC7815626 32813049

[20] V. Salsi , F. Magdinier , and R. Tupler , “Does DNA Methylation Matter in FSHD?,” Genes 11 (2020): 258. 10.3390/genes11030258 PMC7140823 32121044

[21] L. Gérard , M. Delourme , C. Tardy , et al., “SMCHD1 Genetic Variants in Type 2 Facioscapulohumeral Dystrophy and Challenges in Predicting Pathogenicity and Disease Penetrance,” European Journal of Human Genetics 33 (2025): 784–792. 10.1038/s41431-024-01781-x PMC12185756 39725690

[22] O. Schreiber , P. Schneiderat , W. Kress , et al., “Facioscapulohumeral Muscular Dystrophy and Charcot‐Marie‐Tooth Neuropathy 1A—Evidence for “Double Trouble” Overlapping Syndromes,” BMC Medical Genetics 14 (2013): 92. 10.1186/1471-2350-14-92 PMC3848428 24041033

[23] M. Butz , M. C. Koch , W. Muller‐Felber , R. J. L. F. Lemmers , S. M. van der Maarel , and H. Schreiber , “Facioscapulohumeral Muscular Dystrophy,” Journal of Neurology 250 (2003): 932–937. 10.1007/s00415-003-1116-y 12928911

[24] Z. Wang , L. Qiua , M. Lin , et al., “Prevalence and Disease Progression of Genetically‐Confirmed Facioscapulohumeral Muscular Dystrophy Type 1 (FSHD1) in China Between 2001 and 2020: A Nationwide Population‐Based Study,” Lancet Regional Health–Western Pacific 18 (2022): 100323. 10.1016/j.lanwpc.2021.100323 PMC8671729 35024656

[25] G. Ricci , F. Torri , L. Ruggiero , et al., “Describing Phenotypes in FSHD: An Update of the Comprehensive Clinical Evaluation Form,” Neurological Sciences 46 (2025): 4633–4643. 10.1007/s10072-025-08276-7 40506648

[26] V. Salsi , M. Chiara , S. Pini , et al., “A Human Pan‐Genomic Analysis Reconfigures the Genetic and Epigenetic Make Up of Facioscapulohumeral Muscular Dystrophy,” preprint, medRxiv, August 2, 2023, 10.1101/2023.06.13.23291337.

